# Prediction of lncRNA–Protein Interactions via the Multiple Information Integration

**DOI:** 10.3389/fbioe.2021.647113

**Published:** 2021-02-25

**Authors:** Yifan Chen, Xiangzheng Fu, Zejun Li, Li Peng, Linlin Zhuo

**Affiliations:** ^1^College of Information Science and Engineering, Hunan University, Changsha, China; ^2^School of Computer and Information Science, Hunan Institute of Technology, Hengyang, China; ^3^College of Computer Science and Engineering, Hunan University of Science and Technology, Xiangtan, China; ^4^Department of Mathematics and Information Engineering, Wenzhou University Oujiang College, Wenzhou, China

**Keywords:** feature representation, mutual information, structure analysis, support vector machine, lncRNA protein interactions

## Abstract

The long non-coding RNA (lncRNA)–protein interaction plays an important role in the post-transcriptional gene regulation, such as RNA splicing, translation, signaling, and the development of complex diseases. The related research on the prediction of lncRNA–protein interaction relationship is beneficial in the excavation and the discovery of the mechanism of lncRNA function and action occurrence, which are important. Traditional experimental methods for detecting lncRNA–protein interactions are expensive and time-consuming. Therefore, computational methods provide many effective strategies to deal with this problem. In recent years, most computational methods only use the information of the lncRNA–lncRNA or the protein–protein similarity and cannot fully capture all features to identify their interactions. In this paper, we propose a novel computational model for the lncRNA–protein prediction on the basis of machine learning methods. First, a feature method is proposed for representing the information of the network topological properties of lncRNA and protein interactions. The basic composition feature information and evolutionary information based on protein, the lncRNA sequence feature information, and the lncRNA expression profile information are extracted. Finally, the above feature information is fused, and the optimized feature vector is used with the recursive feature elimination algorithm. The optimized feature vectors are input to the support vector machine (SVM) model. Experimental results show that the proposed method has good effectiveness and accuracy in the lncRNA–protein interaction prediction.

## Introduction

Long non-coding RNA (lncRNA)–protein interactions play an important role in the post-transcriptional gene regulation, polyadenylation, splicing, and translation, and predicting lncRNA–protein interactions helps to understand lncRNA-related activities (Mittal et al., 2009; Ray et al., 2013). With the rapid advancement of high-throughput technologies and the rapid increase of lncRNA and protein sequence data, predicting lncRNA–protein interactions by traditional biological experimental approaches, such as RNA-pulldown, RNA immunoprecipitation, and other biological experiments, is expensive and time-consuming. In recent years, computational methods, especially machine learning methods, have been widely used in the field of bioinformatics. For example, Link prediction paradigms have been used to predict drug targets (Munir et al., 2019; Srivastava et al., 2019; Zeng et al., 2019, 2020; Ru et al., 2020; Wang et al., 2020), enhancer promoter interactions (Hong et al., 2019; Cai et al., 2020a), disease genes (Zeng et al., 2017a; Ji et al., 2019; Kuang et al., 2019; Wang et al., 2019; Peng et al., 2020), link prediction (Xiao et al., 2018, 2019, 2020), circular RNAs (Zeng et al., 2017b; Xiao et al., 2019), microRNAs (miRNAs) (Xiao et al., 2018, 2020; Zeng et al., 2018; Hajieghrari et al., 2019; Jeyaram et al., 2019; Zhang X. et al., 2019), and peptide recognition (Bai et al., 2019; Cai et al., 2020b; Fu et al., 2020; Zhang and Zou, 2020). In addition, computational intelligence such as evolutionary algorithms (Song et al., 2020a,b) and unsupervised learning (Lambrou et al., 2019; Noureen et al., 2019; Zhang L. et al., 2019; Zou et al., 2020) can be applied to the field of bioinformatics. Given the efficient performance of machine learning methods in predicting lncRNA–protein interactions, the number of researchers considering machine learning methods as the first choice for predicting lncRNA–protein interactions have been increasing.

The general process of machine learning methods for predicting lncRNA–protein interactions is as follows. First, raw lncRNA and protein data are mined and analyzed separately to extract the characteristic information of lncRNA and protein. Algorithms are then designed to compute the lncRNA–protein interactions and obtain their relationships. Finally, prediction results are verified and can be used to guide biological experiments in reverse, which can reduce the cost of biological experiments and improve the efficiency of research. Currently, machine learning-based methods for predicting lncRNA–protein interactions can be divided into two main categories.

(1) Construction of prediction models on the basis of lncRNA and protein features. The feature information of lncRNA and protein can be extracted using feature extraction methods based on sequence information, structure, and various physicochemical properties, which are fused to construct feature vectors. Feature vectors are fed into machine learning classification algorithms to construct prediction models for lncRNA–protein interaction relationships. Bellucci et al. (2011) have proposed the catRAPID model for predicting lncRNA–protein interactions, which combines the protein molecular secondary structure and the position information and extracts and inputs more than 100 dimensions of feature information from protein and non-coding RNA into the random forest (RF) and the support vector machine (SVM) to train the prediction model. Muppirala et al. (2011) have developed the RPISeq method, which utilizes only lncRNA and protein sequence information and uses SVM and RF classifiers to construct a model for the prediction of lncRNA–protein association interactions. Wang et al. (2013) have applied the plain Bayesian to construct prediction models for predicting lncRNA–protein interactions on the basis of the study of Lu et al. (2013) have proposed a method called the lncPro, which extracts amino acid and nucleotide sequence information and applies the Fisher's linear discriminant method to construct the prediction model. Subsequently, Suresh et al. (2015) have proposed the RPI–Pred method, which extracts the sequence and the structural feature information of lncRNAs and proteins and the high-order 3D structural features of proteins to construct prediction models. However, the low conserved nature of lncRNA sequences makes the prediction algorithm based on lncRNA and protein feature information perform poorly in terms of accuracy and the prediction efficiency and needs to be enhanced.

(2) Heterogeneous network-based prediction model. Given the development of related experimental techniques and the accumulation of research results in the field of lncRNA, many lncRNA–protein interaction relationships have been experimentally confirmed, and researchers have successively proposed many network-based prediction algorithms to study the interaction relationships between lncRNAs and proteins. Li et al. (2015) have constructed lncRNA and protein similarity networks and combined the existing lncRNA and protein interaction data to predict unknown lncRNA–protein interaction relationships and proposed a heterogeneous network-based method called the LPIHN. The LPIHN method predicts unknown lncRNA–protein interaction relationships by constructing a heterogeneous network with the restart random walk (RWR) implemented on the constructed network to predict novel lncRNA–protein associations. Ge et al. (2016) have introduced a network dichotomy method called the LPBNI. This method performs a resource allocation procedure in the lncRNA–protein dichotomous network to evaluate candidate proteins for each lncRNA for the prediction of interaction deletions. Hu et al. (2017) have proposed a semisupervised method called the LPI–ETSLP, which reveals lncRNA–protein correlations and does not require negative samples. On the one hand, the number of known action–relationship pairs is sparse compared with the huge number of lncRNAs and proteins and directly affects the network construction and the performance of the network link prediction. On the other hand, lncRNAs or proteins with only one action–relationship in which the data behave as isolated nodes in the network and most algorithms based on network link prediction cannot effectively predict isolated nodes.

Based on the above analysis, this paper proposes a multifeature information fusion method based on lncRNA and protein sequence features and heterogeneous network topological features to predict lncRNA and protein interaction relationships. First, a novel feature extraction method based on the topological feature information of lncRNA and protein heterogeneous networks is proposed to extract the topological network features of lncRNA and protein, lncRNA sequence mutual information, the basic statistical information of lncRNA sequence bases and lncRNA expression profile features, and the evolutionary information and the composition–transition–distribution (CTD) feature information of protein sequences. Then, the above features are fused, and the fused feature information are input into the SVM to train and construct the lncRNA–protein prediction model.

## Materials and Methods

### Framework of the Proposed Method

**Figure 1:**
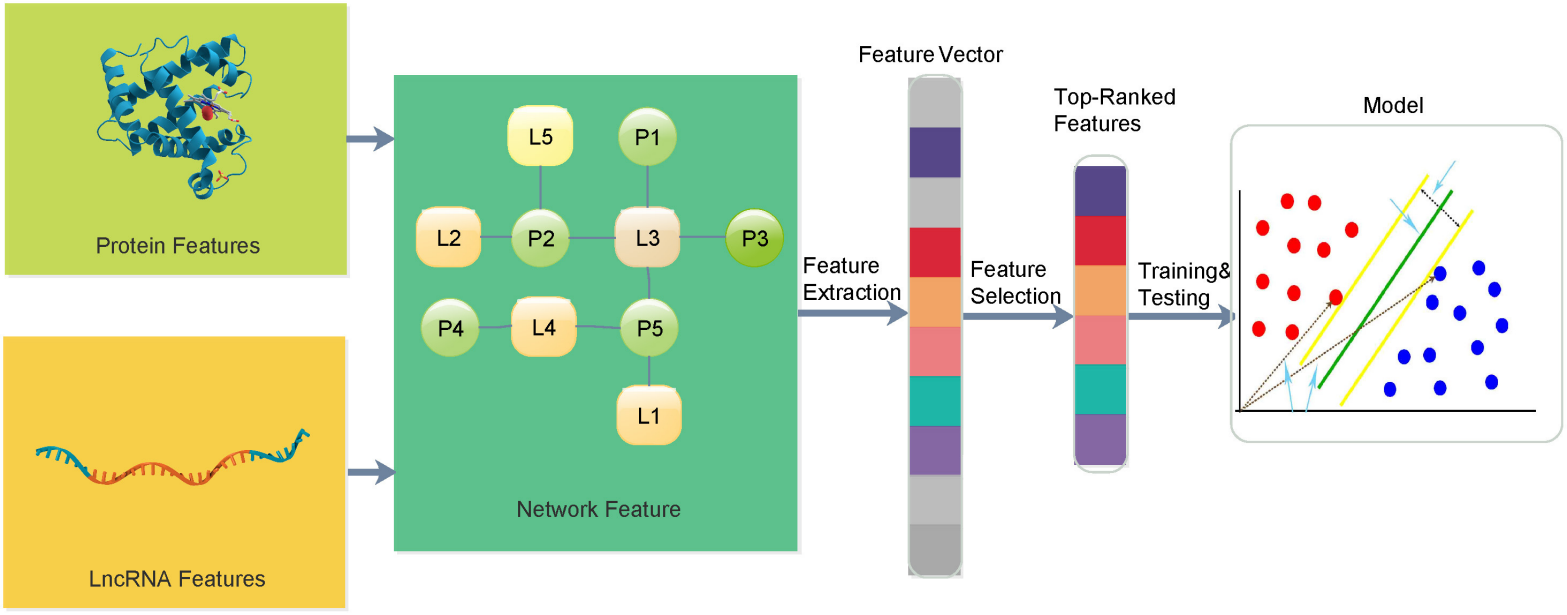
The overall framework of the proposed method for lncRNA–protein interactions.

In this paper, we propose a multi-information fusion-based lncRNA–protein association prediction model consisting of three main phases, namely, (1) dataset preparation, (2) feature extraction and optimization, and (3) model training and prediction. In the dataset preparation, candidate lncRNA and protein sequences and their interaction data are usually collected from validated databases and related literature. Good training and test sets are usually required to build a high-quality prediction model. The training set is used for model training, and the test set is used to verify the transferability and the reliability of the training model. In the feature extraction and optimization, lncRNA and protein topological network features are proposed, and the protein sequence, Position Specific Scoring Matrix (PSSM), lncRNA sequence, and lncRNA expression spectrum features are extracted. Feature vectors are usually optimized by removing some irrelevant features to improve the performance of the feature information. In the model training and prediction, the SVM is used to train the input training set, and the grid search provides SVM training parameters for the construction of the training model. The prediction is performed on the given set of prediction vectors. The overall framework of the entire lncRNA–protein association prediction model is shown in Figure 1.

### Datasets

With the development of high-throughput sequencing technologies, many public databases are available for scientists to study lncRNA–protein interactions. The NPInter database includes experimentally validated information on interactions between non-coding RNAs and other biomolecules (e.g., proteins, RNAs, and genomic DNA). The NONCODE (Liu et al., 2005) database is a comprehensive annotation database covering all types of non-coding RNAs except tRNAs and rRNAs. The NONCODE4.0 database contains 141,353 lncRNA sequence data, covering the lncRNA sequence data required in this paper. The UniProt database (Consortium, 2018) can provide the protein sequence data required in this paper. Through the abovementioned public databases, the datasets required to study lncRNA–protein interactions can be obtained and may help in the conduct of the study.

The acquisition and the preprocessing of datasets usually consist of two main steps, i.e., candidate data collection and invalid data rejection. (1) Candidate data collection, human lncRNA, and its association term data are extracted from the NPInter V2.0 database (Yuan et al., 2013; Hao et al., 2016), and 4,870 pairs of experimentally identified lncRNA–protein interaction datasets, which include 1,114 lncRNAs and 96 proteins, are obtained. Then, the lncRNA sequence information is obtained from the NONCODE 4.0 database, and the protein sequence information is obtained from the UniProt database. (2) Eliminate invalid data; since a few lncRNA sequence data are not available in some candidate datasets, proteins and lncRNAs with unavailable sequence information should be removed. In addition, some lncRNAs that only interact or are related to one protein or proteins that only interact or are related to one lncRNA have usually low correlation and potentially noisy information. Therefore, such data are excluded.

A dataset containing 4,158 lncRNA–protein interactions (including 990 lncRNAs and 27 proteins) is constructed in this paper through the above data processing steps.

### Features Extraction

In this paper, five types of feature information, namely, lncRNA–protein network topology features, protein evolution information (Shao et al., 2020), protein sequence features (Liu et al., 2019), lncRNA sequence features, and lncRNA expression profile feature information, are extracted for the lncRNA–protein association prediction.

#### lncRNA–Protein Network Topology Features

The lncRNA–protein network can be regarded as a heterogeneous undirected graph. Suppose that the lncRNA–protein network contains *N* lncRNAs and *M* proteins and that the sets of lncRNAs and proteins are denoted by *L* and *P*, respectively, then *L* = {*l*_1_, *l*_2_, *l*_3_, …, *l*_*N*_}, and *P* = {*p*_1_, *p*_2_, *p*_3_, …, *p*_*M*_}. The set of edges *E* of this bipartite graph is denoted by *E* = {*e*_*ij*_ | *l*_*i*_ ∈ *L, p*_*j*_ ∈ *P, e*_*ij*_ = *e*_*ji*_}.

If any node *l*_*i*_ and *p*_*j*_ have an interaction, then *e*_*ij*_ = 1, and vice versa *e*_*ij*_ = 0. The interaction feature *L*_*ij*_ between any lncRNA node *l*_*i*_ and protein node *p*_*j*_ is denoted as the set of edge values of node *l*_*i*_ and all other protein nodes except node *p*_*j*_, i.e., *e*_*ij*_ ∉ *L*_*ij*_,*L*_*ij*_ = {*e*_*i*1_, *e*_*ij*−1_, *e*_*ij*+1_, …, *e*_*iM*_}. Similarly, the interaction feature *P*_*ji*_ between any protein node *p*_*j*_ and protein node *l*_*i*_ is denoted as the set of edge values of node *p*_*j*_ and all other lncRNAs nodes except node *l*_*j*_. Then, *e*_*ji*_ ∉ *P*_*ji*_,*P*_*ji*_ = {*e*_*j*1_, *e*_*j i*−1_, *e*_*j i*+1_, …, *e*_*jN*_ }.

The lncRNA–protein network topology is characterized as:s

(1) $$\begin{array}{l} {\text{LPNet}}_{ij}=L_{ij}\cup P_{ji},\;i=1,\ldots ,N,j=1,\ldots ,M. \end{array}$$

As a result, we can obtain 1,015-dimensional network features.

#### Protein Evolutionary Feature Information

The protein evolutionary feature information is extracted using our previously proposed K-PSSM-composition method (Fu et al., 2018). The K-PSSM-composition feature extraction method is derived from the PSSM-composition feature extraction method. The PSSM-composition, which is proposed by Sharma et al. (2015), is used to extract protein sequence features for the prediction of the protein subcellular localization. The PSSM-composition feature extraction method can mine the evolutionary information of protein sequences but loses the mutual information between 20 amino acid residues and the local information of protein sequences. For this reason, we propose the K-PSSM-composition feature method to alleviate the above problems. In this paper, we have applied the K-PSSM-composition method to extract features from the obtained protein sequence data for the collection of the protein evolutionary feature information. The K-PSSM-composition feature is calculated as shown below.

(2) $$\begin{array}{l} K_{-}PSSM_{-}composition\\ ={\left[PSSM_{{}_{-}}com\left(1\right),\ldots ,PSSM_{{}_{-}}com\left(\lambda \right)\right]}_{1\times \left(400*k\right)} \end{array}$$

Here, λ = 1, …*K*; *PSSM*_*com*(λ) denotes the submatrix features, the calculation of which is shown in Equation (3)

(3) $$\begin{array}{l} PSSM_{-}\text{com}\left(\lambda \right)={\left[F^{A},F^{R},\ldots ,F^{\varphi }\right]}_{1\times 400} \end{array}$$

Here, φ denotes the 20 amino acid residues {A, C, D, E, F, G, H, I, K, L, M, N, P, Q, R, S, T, V, W, Y}. *F*^φ^ represents the row sum of amino acid residues in the sub-PSSM matrix. In this study, *k* = 1; thus, we obtain a total of 400 dimensional features.

#### Protein Sequence Feature Information

In this paper, we have used the CTD (Cai et al., 2003) to extract protein sequence features, which represent the distribution patterns of specific structural or physicochemical properties in a protein or peptide sequence. Twenty amino acids are divided into three groups on the basis of different amino acid properties and represented by three feature descriptors, namely, composition (C), transition (T), and distribution (D). C denotes the percentage frequency of a specific set of amino acid properties in the calculated protein sequence, T depicts the percentage frequency of amino acids characterizing a specific property followed by another property, and D denotes the amino acid fragment describing a specific property of the whole protein sequence. Thirteen physicochemical properties have been used to calculate CTD features. Here, we use the iFeature (Chen et al., 2018) to set default parameters to extract CTD feature information and obtained a total of 504 dimensional features.

#### lncRNA Sequence Features

The extracted lncRNA sequence feature information contains two categories, namely, the lncRNA sequence mutual and the base compositional feature information. The lncRNA sequence mutual information is extracted using our previously proposed PSFMI feature extraction method (Fu et al., 2019) by using the entropy and the mutual information to calculate the interdependence between two bases on a given lncRNA sequence. Specifically, the 3- and the 2-gram mutual information (MI) are calculated as the characteristic information of a given lncRNA sequence.

In this study, we used entropy and MI to calculate the interdependence between bases on a given lncRNA sequence. Specifically, the 3-gram MI and the 2-gram MI were calculated separately as the characteristic information of the given lncRNA sequences. The procedure of the 3-gram triplet mutual information calculation is shown in Equation (4).

(4) $$\begin{array}{l} MI\left(x,y,z\right)=MI\left(x,y\right)-MI\left(x,y|z\right) \end{array}$$

Here *x, y*, and *z* denote three bases that are consecutively adjacent to each other, and the equations for the calculation of MI(*x, y*) and conditional mutual information MI(*x, y*|*z*) are as follows.

(5) $$\begin{array}{l} MI\left(\text{x},\text{y}|\text{z}\right)=H\left(\text{x}|\text{z}\right)-\text{H}\left(\text{x}|\text{y},\text{z}\right) \end{array}$$

(6) $$\begin{array}{l} MI\left(\text{x},\text{ y}\right)=p{\left(\text{x},\text{y}\right)}^{*}\text{ log}\left(\frac{p\left(\text{x},\text{ y}\right)}{p\left(\text{x}\right)*p\left(\text{y}\right)}\right) \end{array}$$

(7) $$\begin{array}{l} MI\left(\text{x},\text{ y}\right)=MI\left(\text{y},\text{ x}\right) \end{array}$$

Where *H*(*x*|*z*) and *H*(*x*|*y, z*) are calculated as follows:

(8) $$\begin{array}{l} H\left(\text{x}\right)=p{\left(\text{x}\right)}^{*}\log\left(p\left(\text{x}\right)\right) \end{array}$$

(9) $$\begin{array}{l} H\left(\text{x}|\text{z}\right)=-\frac{p\left(\text{x},\text{ z}\right)}{p\left(\text{z}\right)}\log\left(\frac{p\left(\text{x},\text{ z}\right)}{p\left(\text{z}\right)}\right) \end{array}$$

(10) $$\begin{array}{l} H\left(\text{x}|\text{y},\text{z}\right)=-\frac{p\left(\text{x},\text{ y},\text{ z}\right)}{p\left(\text{y},\text{ z}\right)}\log\left(\frac{p\left(\text{x},\text{ y},\text{ z}\right)}{p\left(\text{y},\text{ z}\right)}\right) \end{array}$$

Where *p*(*x*) denotes the frequency of occurrence of base *x* in the lncRNA sequence, *p*(*x, y*) denotes the frequency of occurrence of 2 grams of bases *x* and *y* in the lncRNA sequence, and *p*(*x, y, z*) denotes the frequency of occurrence of 3 grams of bases *x, y*, and *z* in the lncRNA sequence. The values of *p*(*x*), *p*(*x, y*), and *p*(*x, y, z*) can be calculated by Equations (11)–(13) as follows.

(11) $$\begin{array}{l} p\left(\text{x}\right)=\frac{{\text{N}}_{\text{x}}+\varepsilon }{\text{L}} \end{array}$$

(12) $$\begin{array}{l} p\left(\text{x},\text{ y}\right)=\frac{{\text{N}}_{\text{xy}}+\varepsilon }{\text{L}-1} \end{array}$$

(13) $$\begin{array}{l} p\left(\text{x},\text{ y},\text{ z}\right)=\frac{{\text{N}}_{\text{xyz}}+\varepsilon }{\text{L}-2} \end{array}$$

Here, *N*_*x*_ denotes the number of bases *x* that appear in the pre-miRNA sequence and *L* is the length of the given lncRNA sequence. The ε in Equations (11–13), denoting a very small positive real number, is used to avoid using 0 as the denominator.

For the lncRNA base composition feature information, given any lncRNA sequence, we have calculated the percentage of 4 nucleotide (i.e., A, C, G, and T) and 16 dinucleotide (e.g., AA, AG, and AC) types in each lncRNA sequence separately and obtained 20-dimensional feature vectors. The lncRNA sequence mutual information and the lncRNA base composition feature information have 19 and 16 dimensions, respectively. Thus, the total number of lncRNA sequence feature dimensions is 35; i.e., the dimensionality of the feature vector is 35 dimensions.

#### lncRNA Expression Profile Features

In this paper, we have obtained the lncRNA expression profile information from the NONCODE4.0 database, which contains 170,601 lncRNA expression profile data. The expression profiles describe the expression of lncRNAs in 24 types of human tissues or cells. Thus, the lncRNA expression profile features contain 24-dimensional feature vectors.

By the above analysis, we can extract a total of 1,978 (1,015 + 400 + 504 + 35 + 24) dimensional features obtained.

#### Feature Optimization

The feature space of lncRNA–protein interactions consists of five features, namely, lncRNA–protein network topology, lncRNA sequence, lncRNA expression profile, protein CTD information, and protein sequence evolution information features. Compared with individual features, the fusion of multiple features can capture increased sequence information, which leads to improved prediction performance. However, the fusion of multiple features produces a high-dimensional redundant feature and may lead to problems, such as excessive training time and bias in performance. Therefore, in this paper, we have used the SVM Recursive Feature Elimination (SVM-RFE) and Correlation Bias Reduction (CBR) (Yan and Zhang, 2015) to optimize the feature set.

The SVM-RFE algorithm proposed by Tolosi and Lengauer (2011) has been successfully applied to many system biology problems. The CBR algorithm has been used to reduce potential biases in linear and non-linear SVM-RFE. In this study, we use the algorithm SVM-RFE + CBR (Yan and Zhang, 2015), which consists of a combination of SVM-RFE and CBR, to optimize the feature vectors. The specific process is as follows: first, all features are ranked using SVM-RFE + CBR (Yan and Zhang, 2015) to select a set of features with the top score; second, the selected features are reorganized into new, ordered features; and finally, these new features are fed into the predictive classifier to generate a training model. Thus, we can obtain the ranked list of features through the SVM-RFE and CBR and select a set of top-ranked feature information to enable the optimal selection of features.

In the SVM-RFE + CBR method, we used the following parameters: kerType, rfeC, rfeG, useCBR, Rth. The values and descriptions of these parameters are shown in Table 1. The rest of the required parameters use the default settings of the SVM-RFE + CBR method.

**Table 1:** Parameter description in the SVM-RFE + CBR method.

**Parameter**	**Value**	**Describe**
kerType	2	Kernel type, see libsvm. linear: 0; rbf:2
rfeC	16	Parameter C in SVM training
rfeG	0.0078	Parameter g in SVM training
useCBR	True	Whether or not use CBR
Rth	0.9	Corrcoef threshold for highly corr features

#### Classification Algorithm

In this paper, we choose SVM as the classifier to build the prediction model. Specifically, the open source Library of Support Vector Machines (LIBSVM) is used for model training and construction. The LIBSVM toolbox can be downloaded for free at http://www.csie.ntu.edu.tw/~cjlin/libsvm. We integrated the toolbox in the Matrix Laboratory (MATLAB) workspace to build predictive models. The specific form of the kernel function has a large impact on the performance of the SVM. The Gaussian radial basis kernel function (RBF) has good results for non-linear classification and is widely used for bioinformatics classification; therefore, we choose RBF as the kernel function for SVM. A grid search based on five-fold cross-validation was applied to optimize the SVM parameters γ and the penalty parameter C. The grid search yielded the optimal C = 256 and γ = 0.002 set as their values.

### Measurements

Several measures were used to evaluate the performance of the lncRNA–protein interaction prediction method comprehensively (Jin et al., 2019; Manavalan et al., 2019; Manayalan et al., 2019; Su et al., 2019a,b, 2020a,b; Qiang et al., 2020). The receiver operating characteristic curve was based on specificity and sensitivity. The area under the receiver operator characteristic curve (AUC) and the area under precision-recall curve (AUPR) were used as evaluation metrics (Wei et al., 2014, 2017a,b; Tang et al., 2020). The AUC provided a measure of classifier performance. A high AUC value indicated improved performance of the classifier. However, for class imbalance problems, the AUPR penalizes false positives in the evaluation and is more suitable than the AUC. In addition, the Matthew correlation coefficient (MCC) was used to assess the prediction performance. The MCC considered true and false and positive and negative and was usually a balanced measure that could be used even if these classes had different sizes. Sensitivity (SE), specificity (SP), precision (PR), accuracy (ACC), and MCC are defined as follows.

(14) $$\begin{array}{l} SE=\frac{TP}{TP+FN} \end{array}$$

(15) $$\begin{array}{l} SP=\frac{TN}{TN+FP} \end{array}$$

(16) $$\begin{array}{l} PR=\frac{TP}{TP+FP} \end{array}$$

(17) $$\begin{array}{l} F1-score=2\times \frac{SE\times PR}{SE+PR} \end{array}$$

(18) $$\begin{array}{l} ACC=\frac{TP+TN}{TP+FP+TN+FN} \end{array}$$

(19) $$\begin{array}{l} MCC=\frac{TP\times TN-FP\times FN}{\sqrt{\left(TP+FN\right)\left(TN+FP\right)\left(TP+FP\right)\left(TN+FN\right)}} \end{array}$$

TP, TN, FP, and FN indicate the number of true positives, true negatives, false positives, and false negatives, respectively.

## Results and Discussion

### Analysis of the Effect of Different Feature Information Subsets on the Experimental Performance

The effect of different feature subsets on the experimental performance was analyzed to evaluate the effect of different feature information on the lncRNA–protein prediction performance. We compared each feature subset and their two-by-two combinations on the benchmark dataset separately.

The lncRNA sequence and the lncRNA expression profile features had feature vector dimensions of 35 and 24, respectively. These features were combined for the dimensionality of the lncRNA feature information be 59 and named as lRNA features for convenience. The CTD features of protein sequences were 273 dimensions, and the K-PSSM-composition features of protein evolutionary information were 400 dimensions. The CTD and K-PSSM-composition features were combined and named as Pro features. Thus, the Pro features of proteins were 673 dimensions. The lncRNA–protein topological network features were named LDNet features, and their total feature dimension was 1,015 dimensions. Therefore, six subsets of features [i.e., lRNA, Pro, and LDNet and their two-by-two combinations (i.e., lRNA + Pro, lRNA + LDNet, and Pro + LDNet)] were obtained. To evaluate the effect and the importance of each feature subset on the prediction results, this paper uses the SVM classifier to train the prediction model, and the grid search algorithm was employed to adjust the parameters of the SVM so that each feature subset achieves the best accuracy in the same threshold range. Five-fold cross-validation tests were conducted on these six feature subsets. Experimental results are shown in Table 2.

**Table 2:** Performance of different feature subsets on the benchmark dataset.

**Methods**	**ACC (%)**	**SE (%)**	**SP (%)**	**MCC**	**F1 score (%)**	**AUC (%)**	**AUPR (%)**
LDNet	90.56	77.94	97.14	0.603	64.36	89.32	71.10
Pro	85.87	69.19	98.65	0.290	26.61	57.33	27.92
lRNA	84.47	52.91	**99.77**	0.067	2.83	52.29	20.34
lRNA + Pro	86.17	68.11	98.22	0.323	31.79	79.11	47.94
lRNA + LDNet	**90.81**	**78.69**	97.20	**0.615**	**65.52**	**90.99**	**73.75**
CTD + LDNet	90.62	78.25	97.18	0.606	64.62	89.02	71.32

*The best values are shown in boldface*.

The experimental results of the six feature subsets constructed in this paper by five-fold cross-validation tests are shown in Table 2. The ACC, SE, MCC, F score, AUC, and AUPR values of LDNet features were 90.56, 77.94, 0.603, 64.36, 89.32, and 71.10%, respectively, and higher than those of lRNA and Pro features. For the F1 score, AUC, and AUPR metrics, the LDNet features were higher by 37.75, 31.99, and 43.18%, respectively, than the Pro features, which ranked second in these three feature subsets. Therefore, the LDNet features performed the best in the separate experiments for the three feature subsets of LDNet, Pro, and lRNA, which indicated that the LDNet was the best for the lncRNA–protein association prediction because the LDNet was the largest and far exceeded the two other feature subsets.

The ACC, SE, MCC, F score, AUC, and AUPR values for lRNA + LDNet features were 90.81, 78.69, 0.615, 65.52, 90.99, and 73.75%, respectively, and were the maximum values in these six feature subsets (Table 1). The values of these metrics for Pro + LDNet and lRNA + LDNet feature subsets were close. The F1 score, AUC, and AUPR values for the lRNA + Pro feature subset were 31.79, 79.11, and 47.94%, respectively, which were lower than the first two combined features and even lower than the LDNet feature subset. Therefore, the lRNA + LDNet features performed best in predicting lncRNA–protein interactions. Among lRNA and LDNet features, the LDNet was the main decisive feature subset, which also indicated that the lncRNA and protein network topology-based features proposed in this paper had the greatest effect on the prediction performance. In addition, the performance of each feature subset in the two-by-two combination was better than the feature performance value of each feature subset individually.

### Comparison With Existing Approaches

We selected the following six excellent methods for experimental comparison on the benchmark dataset to compare the performance of our proposed method with existing excellent methods. These six methods included IRWNRLPI (Zhao et al., 2018), LPI–ETSLP (Hu et al., 2017), RWR (Kohler et al., 2008), LPBNI (Li et al., 2015), RPISeq–RF (Muppirala et al., 2011), and RPISeq–SVM (Muppirala et al., 2011). The RPISeq–RF and the RPISeq–SVM models are prediction methods that extract and input lncRNA and protein features into RF or SVM predictors, whereas the IRWNRLPI, LPI–ETSLP, RWR, LPBNI, and RPISeq–RF algorithms are prediction methods that are based on heterogeneous networks constructed from lncRNAs and proteins. On the benchmark dataset, a five-fold cross-validation test was performed separately, and four evaluation metrics, namely, ACC, F1 score, AUC, and AUPR, were selected to evaluate the performance of different algorithms. Experimental results are shown in Table 3.

**Table 3:** Comparison of performance with different excellent algorithms.

**Methods**	**ACC (%)**	**F1 score (%)**	**AUC (%)**	**AUPR (%)**
IRWNRLPI	90.09	65.16	**91.50**	71.38
LPI–ETSLP	88.34	59.78	88.76	64.38
RWR	95.36	36.03	83.32	28.93
LPBNI	**95.81**	38.68	85.86	33.06
RPISeq–RF	46.62	14.81	39.49	6.31
RPISeq–SVM	48.23	14.93	39.87	6.98
Our method	90.82	**65.91**	90.97	**74.39**

*The best values are shown in boldface*.

The experimental results of each evaluation index for predicting lncRNA–protein interactions are listed in Table 3. First, we compared the values of AUPR, which were 64.38% (LPI–ETSLP), 28.93% (RWR), 33.06% (LPBNI), 6.31% (RPISeq–RF), 6.98% (RPISeq–SVM), and 71.38% (IRWNRLPI) lower than 74.39% in our method and indicated that our method predicted reliable results.

**Figure 2:**
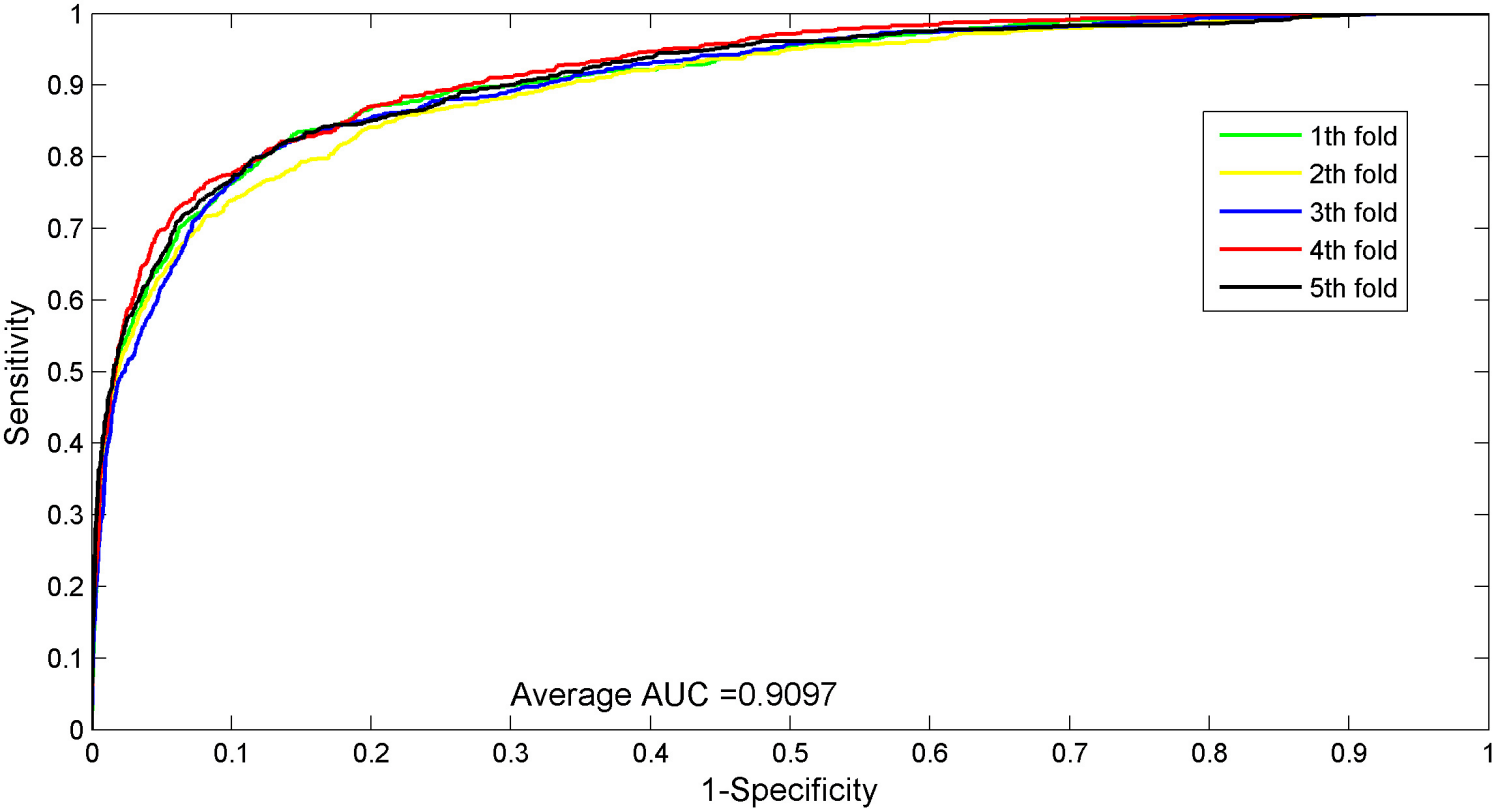
ROC curves for five-fold cross-validation tests of the benchmark dataset.

**Figure 3:**
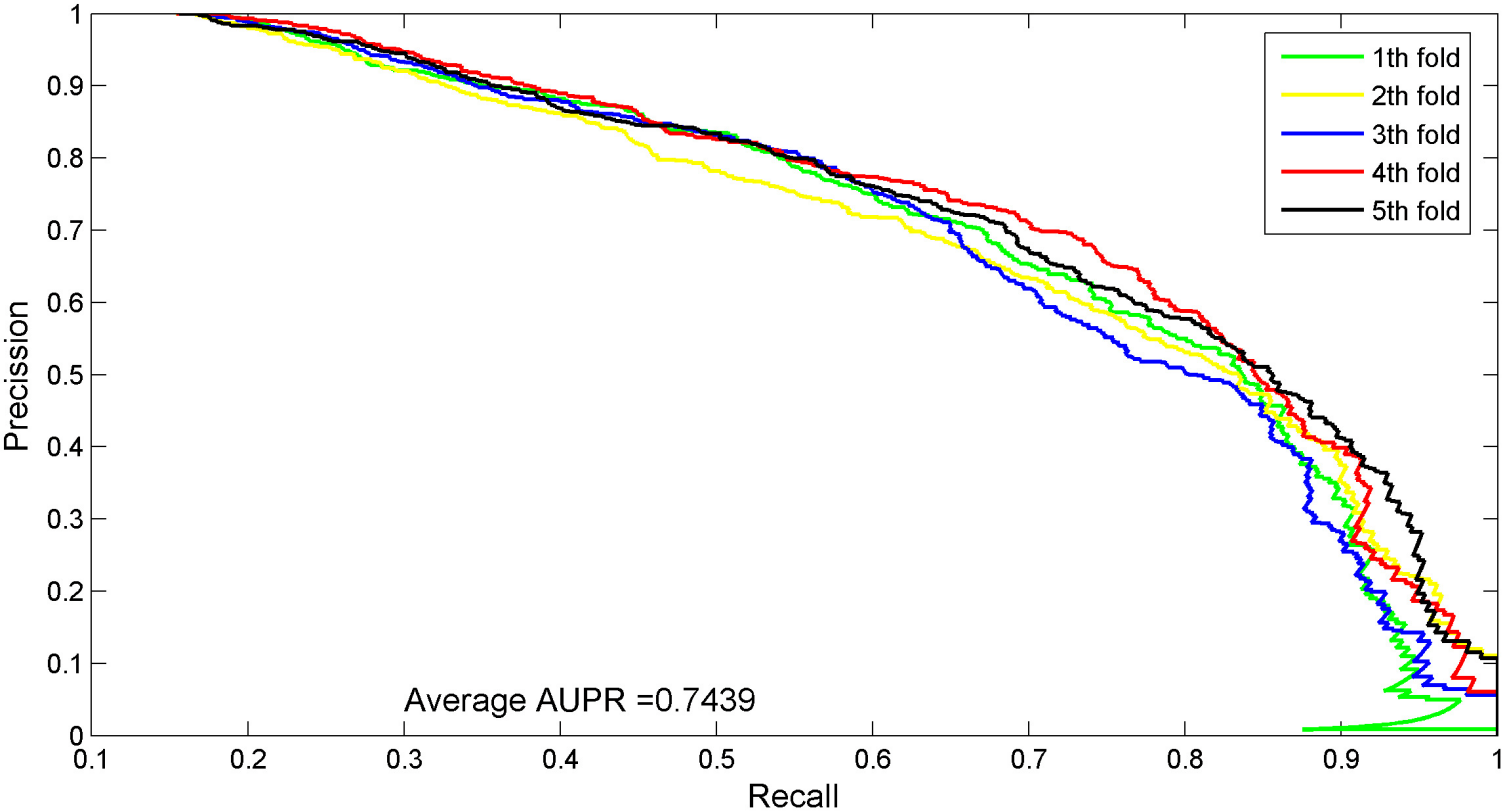
AUPR curves for five-fold cross-validation tests of the benchmark dataset.

The AUC value of our method was 90.97%, which ranked the second among all methods, and was close to the first ranked IRWNRLPI (91.50%) method and 2.21% higher than the third ranked LPI–ETSLP method. These results showed that our method had very good prediction performance. We plotted the curves of AUPR and ROC for the five-fold cross-validation tests to demonstrate the AUPR and the AUC values, respectively (Figures 2, 3).

Next, we further analyzed the ACC and the F1 score values of these prediction models. The ACC of our method was 90.96% smaller than those of RWR (95.36%) and LPBNI (95.81) but better than that of IRWNRLPI (90.09%) because of very few experimentally validated lncRNA–protein interactions, which were far less than the unknown lncRNA–protein association relationships in the benchmark dataset. Therefore, the use of F1 score values to evaluate the performance of different methods than the ACC evaluation was reasonable. The F1 score value of our method was 65.91%, which was the highest among all methods and higher than those of the RWR (36.03%) and the LPBNI (38.68%). Therefore, the combined results of all experiments further demonstrated the good performance of our method in predicting lncRNA–protein associations. Notably, the four evaluation metrics (AUC, AUPR, ACC, and F1 score) of our method, which constructed prediction models on the basis of lncRNA and protein features, were more remarkable than RPISeq–RF and RPISeq–SVM.

## Conclusions

lncRNAs are involved in the regulation of gene expression at the transcriptional level, epigenetics, and other life activity processes by interacting with RNA-binding proteins. Therefore, related research on the prediction of lncRNA–protein interaction relationship is beneficial in the excavation and the discovery of the mechanism of lncRNA function and action occurrence.

In this paper, a computational model for lncRNA–protein interaction relationship prediction based on the multisource information fusion is proposed. A method for representing the topological feature information of the network of lncRNA–protein interactions is proposed. Subsequently, protein evolutionary information, protein CTD sequence information features, lncRNA sequence mutual information features, and lncRNA expression profile information are extracted, and the recursive feature elimination algorithm is used to optimize feature vectors. The obtained optimized feature vectors are fed into SVM to predict lncRNA–protein interactions. Our proposed method is experimentally compared with six excellent lncRNA–protein prediction algorithms by using five-fold cross-validation tests on benchmark datasets, and experimental results show that our proposed method achieves the best performance values in AUPR and F1 score, illustrating the effectiveness and the accuracy of the proposed method in lncRNA–protein association prediction methods.

## Data Availability Statement

The original contributions presented in the study are included in the article/supplementary material, further inquiries can be directed to the corresponding author/s.

## Author Contributions

YC, XF, and LZ conceived the concept of the work. YC, XF, and LP performed the experiments. YC, ZL, and LZ wrote the paper. All authors contributed to the article and approved the submitted version.

## Conflict of Interest

The authors declare that the research was conducted in the absence of any commercial or financial relationships that could be construed as a potential conflict of interest.
